# Value-Based Integrated Care (VBIC) Concept Implementation in a Real-World Setting—Problem-Based Analysis of Barriers and Challenges

**DOI:** 10.3390/healthcare11081110

**Published:** 2023-04-12

**Authors:** Ewa Bandurska, Weronika Ciećko, Marzena Olszewska-Karaban, Iwona Damps-Konstańska, Dominika Szalewska, Piotr Janowiak, Ewa Jassem

**Affiliations:** 1 Center for Competence Development, Integrated Care and e-Health, Medical University of Gdańsk, 80-210 Gdańsk, Poland; 2 Division of Rehabilitation Medicine, Medical University of Gdańsk, 80-210 Gdańsk, Poland; 3 Division of Allergology, Medical University of Gdańsk, 80-210 Gdańsk, Poland; 4 Division of Pulmonology, Medical University of Gdańsk, 80-210 Gdańsk, Poland

**Keywords:** value-based healthcare, value-based integrated care, outcome measures

## Abstract

Healthcare effectiveness measurement and value in health have been common topics in public health literature since 2006 when value-based healthcare (VBHC) was first defined by Porter and Teisberg. The aim of this study was to identify the barriers and challenges related to the implementation of VBHC solutions in the example of Poland. A case presentation was used as a method. The national integrated care programs (KOS-Infarction, POZ-Plus, and comprehensive treatment of chronic wounds) were used to present general challenges, along with the Integrated Care Model (ICM) for patients with advanced chronic obstructive pulmonary disease (COPD), to determine specific difficulties. ICM has been operating since 2012 in Gdańsk and gradually adapted the value-based integrated care (VBIC) approach. An analysis of the available data showed that the greatest difficulties related to the implementation of the VBHC and VBIC concepts are a lack of legal and reimbursement solutions, staff shortages, a lack of educational standards for some members of the multidisciplinary team, and insufficient awareness of the role of integrated care. As the level of preparation to implement VBHC policies varies between individual countries, the conclusions drawn from the experience of ICM and other Polish projects may be a valuable voice in discussion.

## 1. Introduction

Value in healthcare, first defined in 2006 by Michael Porter and Elizabeth Teisberg, is understood currently as a relation between patient-centered outcome measures and the cost of achieving improvement in these results [1]. The proposed definition emphasizes the needs of patients, their quality of life, and the individual approach to patients focused on values that are important from their perspective. In this view, quantitative issues regarding the provision of services (fee for service) are of secondary importance [2].

The broader approach was proposed by Gray, who presents the concept of value in three respects (known as Triple Value):

- Personal—effective care enabling the achievement of patients’ personal goals;
- Allocative—fair distribution of resources between each group of patients;
- Technical—achievement of best possible results with available resources [3].

This concept was primarily introduced in Great Britain; however, in 2019, the European Commission proposed to complete the Triple Value approach with a fourth dimension, which is social value. This dimension is understood as the contribution of healthcare to social participation and connectivity [4]. 

The justification for introducing the value-based healthcare (VBHC) approach is to give more value to patients expectations and put them at the center of interest. Reducing the cost of care matters but is not the most important outcome in the process [5]. Thus, currently, VBHC is most often defined as healthcare that brings the best possible health outcomes for patients at the lowest possible cost of obtaining them [6]. 

From the very beginning, the integration of care has been included in VBHC proposed by Porter; however, VBHC and value-based integrated care (VBIC) are not the same processes. VBIC is not a simple combination of integrated care and VBHC. VBIC was initially defined by Valentijn and Vrijhoef in 2016 as “patients’ achieved outcomes and experience of care in combination with the amount of money spent by providing accessible, comprehensive and coordinated services to a targeted population” [7,8].

To introduce VBIC, the general (population) understanding of health must be included in those elements of VBHC that already exist in the health system. Implementation of VBIC includes, for example, shifting (where possible) from the hospital-centric to more patient-accepted types of care. Availability of home care may result in better adherence to treatment, improved physical activity [9], and better health-related quality of life [10]. It also allows verification of the medicalized vision of what can be called “a therapeutic success”. This success, among others, can include improvement of the functional and laboratory test results of patients, which in VBHC and VBIC are supplemented with data coming directly from the patients. Moreover, VBIC takes into account broader sociological aspects than VBHC. VBIC acts in the context of larger social groups undergoing constant demographic changes and, as a result, presents the changing demand for health services. In addition, in the VBIC model, more attention is given to co-defining value in health and co-creating essential outcome measures in collaboration with people and communities [11]. This translates into the possibility of addressing multimorbidity issues [12]. There is evidence that effective integration of care supports achieving clinically important endpoints [13] as well as improving the patient’s experience of care and reducing its costs [14]. Furthermore, it supports increasing health literacy and quality of life optimization, and improves self-management of the disease [15]. The framework for building VBIC interventions was created based on the Triple Aim model (also known as the Rainbow Model of Integrated Care). Its goals are to improve patients’ experiences of care and general population health, along with healthcare cost reduction. Based on these aims typical for VBHC, specific domains for VBIC were built. These domains include outcome measures, scales, types, and enablers of integration domains. 

In terms of the scale of organization, the macro (universal population), meso (targeted sub-groups), and micro (targeted individuals) levels can be distinguished. Similarly, when analyzing the type of integration, we can identify:

- Macro scale—a comprehensive set of political and systemic arrangements that ensures the provision of care for the general population;
- Meso scale—a partnership established by various stakeholders, mainly for the sake of joint accounting and management that enables the provision of integrated care (IC) to selected groups at risk. It can also be understood as building partnerships between different groups of professionals to better understand their individual roles in providing IC for selected target groups;
- Micro scale—also called patient-centered or clinical care, it is an integrated form of care, but most often limited to individual, specific health condition cases; a single process carried out at a specific time and place.

Functional and normative integration are related to the context of enablers of integration [7]. According to the definition, functional means the existence and use of mechanisms and communication tools that allow for the joint management and settlement of benefits. Normative, in turn, is a typical and coherent cultural and social framework that allows for the acceptance of integrated care and the achievement of the goals set for it [11]. The domains related to outcome measures were used to present progress in the implementation of a program—the Integrated Care Model (further called ICM)—developed in Gdańsk (Pomeranian Province, Poland), which is further discussed as a real-world case of VBIC. 

## 2. Methods

A case study approach was used to determine the barriers and challenges related to the implementation and provision of the Integrated Care Model that would meet the criteria of value-based integrated care (VBIC). This study provides a broad context of national programs with which to identify general barriers and challenges. For discussing specific difficulties, the Integrated Care Model for patients with advanced COPD (further called ICM) was chosen due to its unique elements of: (1) Multiannual functioning in the Polish healthcare system with only single features consistent with the concept of value-based healthcare; (2) Experience with gradual adaptation of the value-based integrated care (VBIC) approach; (3) Offering a wide range of support for patients, including home care, treatment tailored to individual patients’ needs, patient and family education, multidisciplinary team care (including a social worker), and access to a care coordinator. Limitations to this case study include the lack of publications that could be used as comparative material and the fact that individual countries are not equally prepared to implement VBHC. Thus, VBIC models may face slightly different challenges.

## 3. Case Presentation

### 3.1. National Programs 

Health programs which include VBHC elements have been more frequently financed by the Polish public payer (National Health Fund) in the last few years. Nationally coordinated care projects using VBHC elements include, among others, coordinated specialist care for the patient after a heart attack (KOS-Infarction), coordinated care in primary healthcare (POZ Plus), and comprehensive treatment of chronic wounds (KLRP-1 and KLRP-2 services). A particularly interesting example is the KLRP, which introduced modern services with advanced IT tools based on performance bonuses (degree of wound closure) [16]. The KOS-Infarction program includes four modules: hospitalization with the development of a treatment plan and possible invasive treatment, cardiac rehabilitation, electrotherapy, and outpatient cardiac care. The KOS-Infarction program employs a coordinator who supervises the path of patients. What is more, the centers that have their own rehabilitation department are rewarded. Units that participate in the program are obliged to report indicators regarding outcome measures related to the quality of care and treatment results [17]. The results of the implementation of the KOS-Infarction program indicate a lower mortality rate than in the period before the program. Statistical analyses carried out by the Agency for Health Technology Assessment and Tariff System, together with the National Health Fund and the Polish Cardiac Society, showed a 36% relative reduction in the risk of death within a year after a heart attack. The actual annual mortality rate among patients not covered by the KOS-Infarction program was 9.3%, and among those included in the program it was 4.3%. The comparative analysis showed that if the patients actually included in the KOS-Infarction program were not included in it, the annual mortality rate would be 6.1% [18]. Another outcome is shortening the median of waiting time for cardiac rehabilitation (from 53 days to approximately 14 days) and an increase in patient satisfaction with cardiac care. According to 96% of patients, receiving care under the KOS-Infarction program had a positive impact on their health, and 99% felt that the KOS-Infarction program provided a sense of security. One of the weaknesses of the program is its low accessibility. In 2019 (the last year before the pandemic), only 15.5% of eligible patients benefited from comprehensive care, and 8.0% underwent cardiac rehabilitation [19]. 

### 3.2. Own Case—Integrated Care Model for Patients with Advanced COPD

In Poland, in addition to national programs, regional initiatives are also undertaken. Despite being less known, they are characterized by a high degree of advancement in terms of VBHC and, at the same time, serve as an example of integrated care, entering the area of the VBIC concept. One such program is ICM, which is dedicated to patients with chronic obstructive pulmonary disease (COPD), the disease known to be the third leading cause of morbidity and death [20].

**Figure 1:**
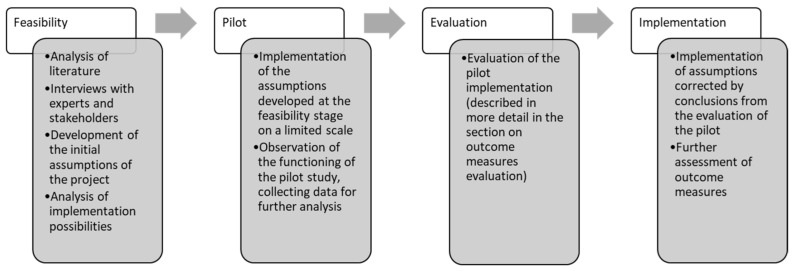
ICM implementation details. Made by the authors. Based on: [22,23,24,25,26,27].

The presented model (ICM) has been operating continuously since 2012 and uses a recommended scheme of implementing services in healthcare (feasibility–pilot–evaluation and implementation, presented in detail in Figure 1) [21].

ICM was presented in detail back in 2012 and later years [28,29]. In the first year of conducting it, patients were asked about their level of acceptance of home visits. The study used a simple scale (very unsatisfied, unsatisfied, neutral, satisfied, very satisfied). Around 93% were very satisfied with the care they received. Patients’ knowledge of COPD was highly unsatisfactory at the beginning of the study but improved significantly (*p* < 0.05) after education provided within ICM [23]. 

ICM is a home-based coordinated intervention for patients suffering from advanced COPD with frequent exacerbations. It is worth underlining that COPD exacerbations are associated with the irreversible loss of lung function [30]. Therefore, all activities of the ICM team aim to counteract patient health decline and to reduce COPD exacerbation and hospitalization rate. The ICM team consists of both medical and non-medical staff, including volunteers. 

Apart from home support, ICM provides the integration of the medical team with social assistance and the assistance of a coordinator. Individual patients’ treatment plans are tailored according to COPS and their individual health needs. In addition, compliance with medical recommendations is monitored and discussed during regular interdisciplinary team meetings. Entry to the program is preceded by qualification conducted by a team of physicians from the Department of Allergology and Pneumonology of the University Clinical Centre in Gdańsk. Consent to enter ICM is obtained from the patient during hospitalization due to an exacerbation of COPD. Afterwards, the coordinator conducts an interview with the patient and arranges appointments with a specialist physician, a psychologist, a physiotherapist, and a dietician. Patients are provided with instructional materials—a book with exercises and a video, including a set of general rehabilitation and breathing exercises. Further, at one of the regular meetings, the team assesses whether including the patient in ICM is advisable. After qualifying, the patient is visited at home by assistants during regular two-hour visits (every two weeks). Assistants check whether the patients have the prescribed drugs and are using them correctly. They also accompany the patients during exercises and assess whether they need the additional support of volunteers or social care institutions. Each visit ends with the preparation of a structured report that is presented to the coordinator at the regular meeting of the team. This report includes information on compliance (including regularity of taking medications), patient needs and satisfaction with care and other important factors that may affect the quality of care. The entire integrated care team meets regularly every 4–6 weeks where they prepare a treatment plan and provide the necessary support for patients. The coordinator organizes the work of the team, enables the patients to stay in contact with the therapeutic team, and ensures contact between the team members. Patients and their families are educated during individual consultations and regular patient gatherings. The topics of the educational meetings focus on the essence of the disease, the principles of the therapy, including the use of individual inhalers, the treatment of exacerbations, the role of rehabilitation, and diet. The team will also look for ways to provide effective non-medical care when the difficult socio-economic situation of the patient is an additional challenge. Participation in ICM ends when the patient’s consent is withdrawn or the disease progresses to the terminal phase, requiring the care of palliative support centers [28].

**Figure 2:**
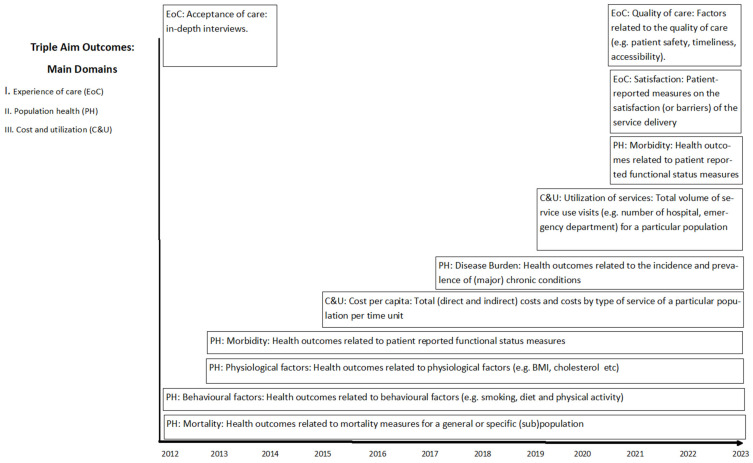
Timeline showing the addition of new dimensions of effectiveness evaluation in ICM. Made by the authors.

ICM is carefully analyzed in terms of effectiveness. The types of outcome measures used to assess ICM have been gradually extended with additional areas [31]. This can be seen on the timeline (Figure 2). The analysis of the effectiveness of ICM started by assessing mainly the objective indicators of the patient’s health measured by clinicians (2012) [32] and then developed into analyses of cost indicators (2015) [26], followed by additional analyses of changes in care utilization and demand (2019) [27] and, finally, beginning in 2022, it started to meet the VBIC criteria in the scope of outcomes measurement, while taking into account PREMs type (Patient Reported Outcome Measures) outcomes.

As can be seen in Table 1, the ICM operation has been evaluated since its inception. The first outcome measure coming from patients of the PROMs type (Patient Reported Outcome Measures) was obtained with a CAT questionnaire. This study has been conducted continuously since the beginning of the model’s operation [23]. From 2022, PREMs type (Patient Reported Experience Measures) outcome measures were added to assess the experiences of patients related to the care received and the impact of COPD on their lives. This study is still ongoing. 

All analyses of costs and changes in demand for health services have been established as before–after type and included two consecutive 6-month periods—in the first 6 months, patients received standard care, followed by integrated care. 

It should be emphasized that all available results clearly indicate that the ICM is a cost-effective intervention, regardless of the type of indicator used in the assessment of effectiveness, which is also presented in Table 2. The exception was a lack of statistically significant reduction in the value of general direct medical costs (costs of all procedures). An in-depth qualitative analysis showed that this was due to a factor that should be considered as positive. After entering the ICM, patients more often used services that were not related to COPD. One reason for this might be that patients felt better and were more concerned about their overall health. They used dental care, dermatologist’s services, or rehabilitation more often than before entering the ICM.

## 4. Discussion

A study by Lewis et al. showed that the instability of state health policy can be a key obstacle to the implementation of integrated care models [12]. This may be one of the reasons why Poland is not fully ready for VBHC and VBIC implementation [33]. Available data from international comparisons show that, although Poland is above average in all VBHC implementation domains, with leaders being, e.g., the Netherlands, some challenges appear [34]. It is also worth emphasizing that the understanding of the concept of “value” as well as the decisions made in the field of state health policy depend on the advancement of the healthcare system in adopting VBHC rules.

The initial attempts to implement projects based on VBHC and VBIC in Poland revealed several barriers and challenges. Recalling the previously described wound treatment services, few service providers are interested in conducting them. In 2021, the KLRP-2 service was delivered by only three healthcare providers in two voivodships in Poland (out of 16). The reasons for this include the high staff and organizational requirements, a lack of adequate financial incentives, and actual (not only declarative) incentives to shift the burden from inpatient to outpatient care. 

There are also many difficulties in conducting ICM. This is mainly due to the lack of a stable source of financing and dedicated forms of payment that would take into account the specificity of integrated care. In addition, the healthcare system is not organizationally prepared to provide patients with advanced COPD with full coverage of their needs. A project is currently underway under the ICM leadership to improve the integration of activities between the GP and the specialists (and the hospital) through the use of an online platform. Using these modern techniques might improve the patient’s “pathway” between medical centers and may increase the availability of different professionals. Barriers to the implementation of integrated care also include the lack of staff, including coordinators and nurses, among others. In particular, the position and role of the coordinator requires formal regulation; so far, it is a function, not a profession. For the purposes of ICM, the authors prepared individual training sessions for all team members, which were carried out during monthly meetings. The ICM coordinator has no medical education, which additionally emphasizes the need to popularize and standardize training in this area. At the moment, there are no systemic solutions in this regard in Poland. Although a few universities train coordinators, the educational offers are very scarce. Additionally, the role of interprofessional communication is seldom present in study programs. It seems that many implementations outpaced the training of personnel.

The barriers identified in Poland based on experience from conducting national programs are systemic and concern various stakeholders, starting with society itself, which must be ready to accept the new form of care and to demonstrate a more active and responsible form of participation in the provision of health services. A good global example of where patients are active in VBHC is their involvement in ICHOM’s development of standard sets of outcome measures [35].

The next group is healthcare providers, who need to engage additional resources to transit to VBHC, which often involves the need to reorganize work and retraining. This may require cutting the provision of many different services off, which raises the risk of fragmentation of care and makes it challenging to implement package payments. It is also associated with the need to perform new activities, e.g., entering data into dedicated registers, measuring new outcome measures, and others [36]. The critical moment is the reorganization of care from a medical specialty approach (e.g., cardiology, ophthalmology, diabetes) to patient-defined care (groups of patients). This requires changes in the coordination of treatment in the patient’s pathway and the creation of multidisciplinary teams responsible for the whole treatment [37,38,39]. A particular challenge is to make an adequate assessment of integrated care systems and to clearly define them. This is a challenge not only in Poland, but also in countries that have been implementing such programs for many years. They indicate, among others, differences in the perception of the role of “outcome measures” and expectations related to cost reduction between healthcare providers as well as local and national actors [12]. It is crucial to develop methods for evaluating integrated care systems based on reliable and generically diverse data (including PROMs and PREMs). This will contribute to a more rational and transparent process of launching integrated care models [40].

Integrated care based on VBHC also requires a change within social care, which must cooperate more widely with, for example, healthcare providers [29,41].

However, key challenges remain in the decision-making area of the government, as a creator of general conditions necessary for implementation of VBHC and VBIC. Above all, it is necessary to remove the remaining legal barriers and actively support the introduction of new value-based reimbursement models. Regarding integrated care itself, it is crucial to remove organizational, financial and legal barriers, and to introduce an incentive system for smaller centers to jointly initiate integrated care in partnership with larger entities. 

It is also necessary to develop the already-active solutions towards the implementation of payments dependent on the outcome. This would require obliging service providers to report outcome measures, creating a national framework for registries in the area of outcome measures dedicated to individual medical conditions and preparing cost reporting standards. All these activities require the refinement of existing and functioning IT systems. 

Some actions are also needed on the part of the public payer. In terms of implementing integrated care, the main one is to introduce incentives, including financial ones, dedicated to service providers who operate in the form of an integrated practice unit (IPU) and support the coordination of patient care throughout the treatment pathway. Concerning the reimbursement process itself, developing reimbursement structures for entire care cycles is still a challenge [37]. 

VBIC remains an important topic that is constantly challenging and which can help optimize healthcare [42]. During the outbreak of the COVID-19 pandemic, it was the VBIC idea that was recommended for organizing vaccine delivery based on the patient’s treatment pathway [43]. Proposals that fit the VBIC concepts are required nowadays as they support care in a broad, horizontal way, focused not on a disease but on the well-being of the patients, supporting the role of family and community and including the cost perspective. Similar actions are being carried out in other countries, but these are not yet standard solutions. The review conducted in 2016 showed that entities implementing VBIC-compliant activities (n = 33) most often operate in the field of mixed domains (58%). Out of the reported outcomes measures, the EoC domain reports quality of care (39%), the PH domain reports different types of outcomes measures (61%), and in the C&U domain most often no outcome was reported (58%) [11]. It is believed that their small number is the main reason for the lack of knowledge on this subject. Currently, this topic is being developed in a variety of areas—VBIC evaluation [44], the use of management concepts [45], and application in specific groups of patients [46]. 

## 5. Limitations and Recommendations for the Future

The presented study has some limitations. The most important is the limited number of publications dealing specifically with VBIC, not VBHC in general. The small number of VBIC programs that are sufficiently well described is considered to be the main reason for the lack of knowledge in this area and presents limited possibilities with which to conduct comparative analyses. ICM, shown in this article as a real world case, operates in a healthcare system that does not fulfill all the principles of VBHC. Therefore, the ICM itself faces some challenges that may not be present in other projects and countries. However, as the degree of preparation for the implementation of the VBHC concept is varied and the discussion on opportunities and challenges is important, the experiences from conducting ICM can be helpful in selecting the most significant goals and ways of solving already-identified problems (such as lack of legal and reimbursement solutions, staff shortages, lack of educational standards for some members of the multidisciplinary team, insufficient knowledge about the role of integrated care, and others). The lessons learned from the multiannual operation of ICM, including the gradual adaptation of the value-based integrated care (VBIC) approach, may be valuable in international discussions on this subject.

## 6. Conclusions

The ICM presented in this article is a rare proof that, even without systemic solutions enabling the implementation of VBHC, it is possible to implement effective projects fitting the VBIC concept. However, they have to face many challenges and difficulties, which are the result of the lack of necessary action on the legislative side, in particular concerning reimbursement methods. The lack of systemic solutions is tantamount to the lack of standards that would help initiate such activities in the future and correct programs already in place. The lessons learned from multiannual operation of ICM, including the gradual adaptation of the value-based integrated care (VBIC) approach, may be valuable guidance on how to build such projects and prioritize activities. It seems that, at the moment, it is critical to clearly embed integrated care in the healthcare system as a separately reimbursed service and to further train staff—especially integrated care coordinators.

## Figures and Tables

**Table 1:** Outcome measures used to evaluate the ICM within the first year of use and the type of instrument. Summary based on publications referring to ICM and internal ICM data [25,26,27,28,32].

Type of Outcome Measure	Subtype of Outcome Measures	Year of Commencement of Measurement	Instrument to Measure Outcome	Notes/Comments
**Group of Results Measures: Population Health (PH)**				
Mortality	Group-specific mortality rate per year	2012	n/a	Information about deaths in group of patients receiving IC.
Behavioral factors	Smoking	2012	n/a	Interview with patients conducted by a doctor based on declarations of patients, saved in patients’ health records.
Behavioral factors	Diet, physical activity, regularity in taking medications	2012	n/a	Interview with patients conducted by social carer, saved in patients’ health records, and reports from home visits.
Physiological factors	BMI, spirometry results, glucose level and others	2012	n/a	Factors are measured as needed during the patient’s visits to the family doctor or specialist doctor, saved in patients’ health records.
Morbidity	Impact of COPD on a person’s life	2012	CAT (the first PROM used to evaluate ICM).	Interview with patients conducted by a doctor or ICM coordinator, saved in patients’ health records.
Morbidity	Health-related quality of life.	2022	EQ-5D-5L	
Morbidity	Depression	2022	HADS-M	
Disease burden	Occurrence of comorbidities in patients included in ICM	2017	n/a	Analysis of EHR of patients included in ICM.
**Group of results measures: Cost and Utilization (C&U)**				
Cost per capita [25,26]	Analysis of direct medical costs (DMC) from the perspective of the public payer	2015	n/a	Analysis based on EHR, data obtained from public payer and financial documents of ICM.
Utilization of services [27]	Analysis of changes in demand for medical services (including exacerbation-related).	2019	n/a	
Utilization of services [27]	CEA analysis using ICER ratio	2019	n/a	
**Group of results measures: Experience of care (EoC)—study in progress**				
Experience of care	Experience of living with COPD and the care received	2022	PREM-C9 (the first PREM used to evaluate ICM).	Interview with patients conducted by a doctor or ICM coordinator

HADS-m—Hospital Anxiety and Depression Scale modified version; EQ-5D-5L—EuroQol-5D-5L; PREM-C9—COPD Patient Experience Questionnaire; PROM—Patient Reported Outcome Measure; PREM—Patient Reported Experience Measure; CEA—Cost Effectiveness Analysis; ICER—Incremental Cost Effectiveness Ratio; BMI—Body Mass Index; CAT—COPD Assessment Test, ICM—Integrated Care Model.

**Table 2:** Available results of economic effectiveness of ICM (including ICER). Summary based on publications referring to ICM [25,26,27].

Type of Outcome Measure with a Reference to the Publication Referring to ICM	Subtype of Outcome Measure	Analysis Result for ICM vs. Standard Care	*p*-Value	Comments
**Cost per capita** [25,26]	DMC general	Reduction in average cost of care associated with replacing standard care with ICM for three groups of costs	0.0791	No significant changes observed.
	DMC COPD related		0.0124	
	DMC exacerbations related		0.0170	
**Cost per capita—CBA analysis** [25]	CBR	The ratio of benefits and costs associated with replacing standard care with ICM for three groups of costs	n/a	Intervention profitable if CBR > 1 (criteria met in all cases).
	NPV	Difference between benefits and costs associated with replacing standard care with ICM for three groups of costs	n/a	Intervention profitable if NPV > 0 (criteria met in all cases).
	ROI	The quotient of the value of net benefits and all costs of implementing a given intervention	n/a	Intervention profitable if ROI > 0 (criteria met in all cases).
**Utilization of services** [27]	Changes in demand	Outpatients visits reduction	0.037	
		Hospitalization and emergency visits (including exacerbation related)	0.033	
		Summary—ambulatory and emergency + hospitalization	0.020	
**Utilization of services** [27]	ICER	Cost effectiveness of avoiding: hospitalizations, exacerbation related hospitalizations, and emergency procedures	n/a	ICER < 0 in each case, which indicates that ICM is profitable in comparison with standard care.

CBA—Cost Benefit analysis; CBR—Cost Benefit Ratio; NPV—Net Present Value; ROI—Return On Investment; DCM—Direct Medical Cost; COPD—Chronic Obstructive Pulmonary Disease. 1 EUR = 4.78 PLN.

## Data Availability

Not applicable.
